# Symptom, functional, and medication overlap between long COVID and fibromyalgia

**DOI:** 10.1016/j.clinsp.2026.101046

**Published:** 2026-07-03

**Authors:** Kevin V. Hackshaw, Michelle M. Osuna-Diaz, Katherine R. Sebastian, Shreya Madhav Nuguri, Silvia de Lamo Castellvi, Lianbo Yu, Zhanna Mikulik, M. Monica Giusti, William Michael Brode, Luis Rodriguez-Saona

**Affiliations:** a Department of Internal Medicine, Division of Rheumatology, Dell Medical School, The University of Texas, Austin, USA; b Department of Internal Medicine, Dell Medical School, The University of Texas, Austin, USA; c Campus Sescelades, Departament d’Enginyeria Química, Universitat Rovira i Virgili, Tarragona, Spain; d Center of Biostatistics and Bioinformatics, The Ohio State University, Columbus, USA; e Department of Internal Medicine, Division of Immunology and Rheumatology, The Ohio State University, USA; f Department of Food Science and Technology, The Ohio State University, Columbus, USA

**Keywords:** Long COVID, PASC, Fibromyalgia, FIQR, SF-36, Central sensitization, Medication burden, Centrally acting agents, Biomarkers

## Abstract

**Background:**

Long COVID (LC/PASC) and Fibromyalgia (FM) share prominent pain, fatigue, and cognitive symptoms and are often difficult to distinguish clinically. The authors compared LC/PASC and FM using harmonized questionnaires assessing symptoms, function, and medication burden to quantify phenotype overlap and inform biomarker development.

**Methods:**

The authors analyzed a harmonized dataset including LC/PASC participants and FM-only comparators. Measures included age, sex, BMI, FIQR/SIQR-equivalent, BDI, CSI, MPQ, VAS pain, medication burden derived from free-text entries, and descriptive SF-36 domains.

**Results:**

The sample included 54 LC/PASC and 889 FM-only visits. LC/PASC participants were older and less often female. Across symptom and function measures, partial overlap was observed, with domain-specific differences. Medication burden was common; FM showed greater centrally acting medication use. SF-36 domains showed broad similarity with domain-specific differences.

**Conclusions:**

In this preliminary, hypothesis-generating comparison, LC/PASC and FM demonstrate partial and domain-specific overlap across questionnaire measures, while differences in medication exposure may influence symptom reporting and limit direct clinical comparisons. These findings support the need for prospective studies integrating objective biomarkers with standardized clinical phenotyping.

## Highlights

• Long COVID and fibromyalgia show overlapping symptom profiles.
• Questionnaires alone poorly distinguish LC/PASC from fibromyalgia.
• Medication burden differs and may confound symptom comparisons.
• Central agents are more common in fibromyalgia cohorts.
• Findings support the need for objective biomarkers in both conditions.

## Introduction

Post-Acute Sequelae of SARS-CoV-2 infection (PASC), often termed Long COVID (LC), is characterized by persistent multisystem symptoms including fatigue, exertional intolerance, cognitive complaints, pain, sleep disturbance, and impaired functioning.^1^, ^2^, ^3^, ^4^, ^5^ Fibromyalgia (FM) is a chronic nociplastic pain disorder marked by widespread pain, sensory amplification, fatigue, cognitive symptoms, and functional impairment.^6^, ^7^, ^8^, ^9^, ^10^ In routine clinical settings ‒ particularly rheumatology and pain clinics ‒ LC and FM can present with highly overlapping symptom profiles, complicating diagnostic classification and potentially obscuring distinct biological mechanisms.^11^, ^12^, ^13^

Patient-reported outcome measures quantify important domains of burden in both conditions. Instruments such as the Revised Fibromyalgia Impact Questionnaire (FIQR), Beck Depression Inventory (BDI), Central Sensitization Inventory (CSI), and McGill Pain Questionnaire (MPQ) are widely used in clinical practice and research, and the SF-36 provides a broad functional profile.^14^, ^15^, ^16^, ^17^, ^18^ However, questionnaire constructs overlap substantially, are sensitive to comorbidity and context, and may be influenced by treatment exposure.^19^^,^^20^

Medication burden is particularly relevant when comparing LC and FM. FM cohorts frequently receive centrally acting therapies (e.g., gabapentinoids, antidepressants, sedative-hypnotics, muscle relaxants, and opioids/tramadol), which may modify symptom reporting, pain quality, and function. LC cohorts, in contrast, may have heterogeneous treatment exposure depending on time since infection and referral pathways.^21^^,^^22^ Differences in medication exposure may therefore influence symptom reporting and complicate interpretation of apparent clinical differences. Finally, emerging biomarker strategies (including metabolomic and spectroscopic approaches) have shown promise for distinguishing clinically similar syndromes when symptom profiles overlap.^23^

Building on this motivation, the authors previously demonstrated that objective metabolic profiling can distinguish clinically similar LC/PASC and FM populations even when symptom-based questionnaires show partial overlap. Using a portable Fourier-Transform Mid-Infrared (FT-MIR) spectroscopic platform combined with chemometric modeling, the authors identified reproducible metabolic fingerprints that differentiated LC/PASC from FM with high sensitivity and specificity, including external validation performance approaching perfect classification. Discriminatory spectral features were localized to biologically plausible regions associated with amino acid, lipid, and carbohydrate metabolism, supporting the presence of distinct underlying biochemical signatures despite overlapping clinical presentations. Importantly, this approach required minimal sample preparation and leveraged portable instrumentation, highlighting the feasibility for clinical translation. These findings underscore the limitations of symptom-based instruments alone and provide a strong mechanistic rationale for integrating objective biomarkers alongside clinical phenotyping when evaluating LC/PASC and FM.^24^

The authors therefore conducted a pragmatic LC/PASC-FM comparison using harmonized clinical characteristics, symptom instruments, SF-36 domains, and medication burden derived from free-text medication reporting to contextualize the degree of clinical overlap that motivates biomarker-based diagnostic and mechanistic stratification.

## Methods

### Data source and analytic sample

The authors analyzed a harmonized clinical dataset composed of individuals with primary FM from two institutions: the University of Texas at Austin and The Ohio State University. Participants with LC/PASC were recruited from the Post COVID-19 Program at the University of Texas Health Austin Clinics under the supervision of Dr. W. Michael Brode, Medical Director of the program. The Post-COVID Program currently has a registry of greater than 500 participants, a waiting list of approximately 150 participants, and approximately 45 referrals per month, with 26 new participants seen each month. Individuals with mixed diagnoses (e.g., FM with LC, FM with RA, etc.) or other disease types (e.g., LC with ME/CFS) were excluded. The FM comparator was analyzed at the visit level (n = 889 clinic evaluations); because some individuals contributed repeat visits, the authors conducted a sensitivity analysis restricted to the first available visit per subject, to assess whether results were robust to independence assumptions. The “FM-only” designation reflects clinical diagnostic labeling and exclusion of known mixed diagnoses (e.g., FM with LC/PASC or other inflammatory conditions) within the harmonized dataset; however, systematic ascertainment of prior SARS-CoV-2 infection was not available for all FM participants.

### Clinical outcomes

Demographics included age, sex, and BMI; symptom burden included FIQR and SIQR.^25^ FIQR was prioritized when both were present; when FIQR was missing, SIQR was treated as FIQR-equivalent. Additional outcomes included BDI, CSI, MPQ total pain score, and Visual Analog Scale (VAS) pain.^26^ Functional impairment was assessed using SF-36 domains.

### Medication ascertainment and burden

The free-text field contained medication names. Medications were parsed using pattern-based matching of common generic and brand names and grouped into clinically meaningful categories. Medication burden was summarized as (i) Total medications identified and (ii) Centrally acting medication class count, defined as exposure to gabapentinoids, antidepressants (SNRIs/TCAs/SSRIs), opioids/tramadol, benzodiazepines, sedative-hypnotics, or muscle relaxants.

### Statistical analysis

The authors report mean ± SD with available n for continuous variables and n (%) for categorical variables. Group comparisons used Welch *t*-tests for continuous variables and chi-square tests for categorical variables. Because some LC/PASC outcomes had limited completion, p-values are reported to aid interpretation, but results are emphasized descriptively where sample size is small. Medication exposure was summarized descriptively and was not incorporated into multivariable adjustment models in the current exploratory analysis.

This study was conducted and reported in accordance with the Strengthening the Reporting of Observational Studies in Epidemiology (STROBE) guidelines.

## Results

Variable-specific missingness was handled descriptively; all analyses report available n per measure, reflecting real-world completion patterns in LC/PASC cohorts.

The analytic sample included 54 LC/PASC visits and 889 FM-only visits. LC/PASC participants were older than FM (Table 1). Across symptom instruments, LC/PASC and FM demonstrated partial overlap in FIQR/SIQR-equivalent impact, depressive symptoms (BDI), Central Sensitization (CSI), Pain Quality (MPQ), and VAS pain (Table 1). To address within-subject dependence from repeat FM evaluations, the authors conducted a sensitivity analysis restricted to first FM visits only, yielding 591 FM first-visit evaluations; results were materially unchanged (Table S1). LC/PASC completion rates varied by instrument, with SF-36 domain data available for 43 of 54 visits and similar patterns of variable-specific completion across other measures. All analyses were therefore conducted using available n for each variable. Because of the limited LC/PASC sample size and incomplete domain completion, SF-36 comparisons are presented descriptively rather than inferentially.

**Table 1:** Clinical characteristics, symptom burden, and medication burden (LC/PASC vs. FM-only).

Measure	LC/PASC	FM-only	SMD (LC-FM)
Age, years	48.4 ± 14.1 (n = 52)	42.5 ± 14.0 (n = 887)	0.42
Female, n (%)	40/54 (74.1%)	832/889 (93.6%)	-0.53
BMI, kg/m^2^	29.7 ± 8.9 (n = 42)	31.5 ± 9.0 (n = 846)	-0.20
FIQR (or SIQR-equivalent)	33.0 ± 20.6 (n = 42)	51.9 ± 19.6 (n = 751)	-0.94
Beck Depression Inventory (BDI)	14.2 ± 5.9 (n = 43)	19.8 ± 10.7 (n = 728)	-0.65
Central Sensitization Inventory (CSI)	55.1 ± 16.9 (n = 43)	61.5 ± 14.5 (n = 722)	-0.41
McGill Pain Questionnaire (MPQ)	83.4 ± 51.8 (n = 43)	91.1 ± 46.3 (n = 718)	-0.16
VAS pain (0–10)	3.4 ± 1.9 (n = 43)	5.6 ± 2.1 (n = 857)	-1.10
Total medications identified (free-text)	1.4 ± 1.5 (n = 54)	2.2 ± 1.5 (n = 889)	-0.57
Centrally acting medication classes (count)	1.2 ± 1.4 (n = 54)	2.0 ± 1.3 (n = 878)	-0.61
Any centrally acting agent, n (%)	38/54 (70.4%)	780/878 (88.8%)	-0.46
≥2 centrally acting agents, n (%)	21/54 (38.9%)	546/878 (62.2%)	-0.47

To further assess the potential impact of missing SF-36 data, the authors descriptively compared LC/PASC participants with complete SF-36 domain data (n = 43) versus those with incomplete SF-36 data (n = 11). Participants with complete versus incomplete SF-36 data were broadly similar with respect to sex distribution (64.3% vs. 63.6% female). Participants with incomplete SF-36 data were somewhat younger (42.9±14.2 vs. 51.9±12.0 years) and demonstrated lower available symptom-impact scores (FIQR/SIQR-equivalent: 32.4±27.0 vs. 48.6±18.1), although interpretation is limited because several additional symptom measures (including BDI, CSI, and MPQ) were unavailable in many participants with incomplete SF-36 data. Given the modest sample size and substantial co-occurring missingness within the incomplete subgroup, these comparisons were interpreted descriptively rather than inferentially. Detailed descriptive comparisons are provided in Table S2.

Medication burden differed between groups. Using free-text medication reporting, FM demonstrated higher centrally acting medication exposure than LC/PASC, including a higher centrally acting medication class count and a greater prevalence of two or more centrally acting agents (Table 1). Category-level comparisons showed differing patterns of commonly used centrally acting medications across conditions (Table 3).

SF-36 domain scores in LC/PASC (43/54 visits with available domain data). Available LC/PASC SF-36 observations demonstrated broad overlap with FM across domains (Table 2).

**Table 2:** SF-36 domain scores (descriptive comparison only due to limited LC/PASC sample size and incomplete domain completion; LC/PASC vs. FM-only).

SF-36 Domain	LC/PASC	FM-only
Physical Functioning	34.4 ± 22.2 (n = 43)	39.3 ± 25.0 (n = 718)
Role Limitations – Physical	4.5 ± 16.4 (n = 43)	11.9 ± 25.7 (n = 715)
Role Limitations – Emotional	36.4 ± 41.8 (n = 43)	31.5 ± 39.1 (n = 715)
Vitality (Energy/Fatigue)	10.7 ± 11.0 (n = 43)	19.2 ± 17.5 (n = 716)
Emotional Well-Being	53.7 ± 18.3 (n = 43)	54.2 ± 21.2 (n = 716)
Social Functioning	44.6 ± 21.8 (n = 43)	49.4 ± 25.6 (n = 716)
Bodily Pain	39.8 ± 20.4 (n = 43)	32.6 ± 20.0 (n = 716)
General Health	29.1 ± 17.9 (n = 43)	31.4 ± 18.8 (n = 718)
Health Change	43.2 ± 31.6 (n = 43)	39.1 ± 28.3 (n = 717)

**Table 3:** Medication categories derived from free-text field (LC/PASC vs. FM-only).

Medication category	LC/PASC n (%)	FM-only n (%)	p-value
Gabapentinoids	18/62 (29.0%)	352/890 (39.6%)	0.132
SNRIs	11/62 (17.7%)	201/890 (22.6%)	0.466
TCAs	9/62 (14.5%)	424/890 (47.6%)	<0.001
SSRIs	12/62 (19.4%)	160/890 (18.0%)	0.919
Opioids / Tramadol	6/62 (9.7%)	148/890 (16.6%)	0.208
Muscle relaxants	6/62 (9.7%)	220/890 (24.7%)	0.011
Benzodiazepines	11/62 (17.7%)	113/890 (12.7%)	0.344
Sedative-hypnotics (Z-drugs)	4/62 (6.5%)	40/890 (4.5%)	0.691
NSAIDs	5/62 (8.1%)	217/890 (24.4%)	0.005
Acetaminophen	4/62 (6.5%)	70/890 (7.9%)	0.876

## Discussion

In this pragmatic clinical comparison, LC/PASC and FM demonstrated partial overlap across commonly used symptom and functional instruments. Accordingly, the interpretation emphasizes both shared symptom domains and clinically meaningful distinctions ‒ particularly in pain intensity, vitality, and medication exposure ‒ rather than portraying overlap as the primary or sole finding. Across FIQR/SIQR-equivalent impact, depressive symptoms, central sensitization, pain quality, and VAS pain, the overall phenotype pattern was consistent with a partial-overlap model rather than clear diagnostic separation based on questionnaires alone. Notably, LC/PASC participants in this cohort reported lower pain intensity, functional impact, and depressive symptom burden than FM participants, indicating that while overlapping symptom domains exist, the two groups are not clinically equivalent in this sample.

Medication burden and centrally acting agents: An important interpretive consideration is that the FM cohort largely reflects a pharmacologically managed clinical population, whereas treatment exposure in LC/PASC is more heterogeneous and often represents an earlier stage of therapeutic intervention. As a result, the observed symptom profiles do not represent untreated disease states, but rather conditions shaped by differing medication environments. Centrally acting agents commonly used in FM ‒ such as antidepressants, gabapentinoids, muscle relaxants, and sedative-hypnotics ‒ can influence pain intensity, fatigue, sleep, and mood reporting. Accordingly, between-group symptom comparisons should be interpreted as contrasts between differently treated populations, not as direct measures of underlying disease severity. These differing real-world pharmacologic treatment environments further underscore the need for objective biomarkers capable of distinguishing biological states even when symptom reporting is shaped by treatment exposure. Incorporating medication information from free-text reporting materially strengthens clinical interpretation. FM demonstrated a higher centrally acting medication burden than LC/PASC, including a higher centrally acting medication class count and a higher prevalence of two or more centrally acting agents. This pattern is consistent with longer-standing pharmacologic management in FM cohorts and is clinically important because centrally acting medications can influence symptom severity ratings, pain quality reporting, fatigue, and perceived function.^27^, ^28^, ^29^ Certain centrally acting or neuromodulatory agents used off-label in fibromyalgia, such as low-dose naltrexone, were not analyzed separately due to heterogeneous dosing and inconsistent documentation in free-text medication records. In summary, medication exposure represents an important interpretive factor that may influence symptom reporting when comparing LC/PASC and FM in real-world datasets. Future studies should incorporate multivariable adjustment for medication exposure and treatment duration. Functional outcomes and SF-36 limitations: Domain scores were available for 43/54 LC visits, enabling descriptive comparisons. LC SF-36 demonstrated broad overlap with FM across domains (Table 2). Remaining missingness and the cross-sectional design still limit inference; prospective LC cohorts should harmonize full-domain SF-36 and FIQR capture within the same individuals to enable integrated phenotype modeling.

Why biomarkers matter: When symptom and function instruments yield limited and inconsistent separation ‒ particularly when symptom reporting may be influenced by differing medication exposure ‒ objective biomarkers may help distinguish overlapping symptom phenotypes, resolve diagnostic ambiguity, identify biological subtypes, and support stratified clinical trials. Metabolic fingerprinting approaches such as portable FT-MIR spectroscopy are attractive for translation because they can provide rapid, low-cost signals that may distinguish clinically similar syndromes even when questionnaire profiles overlap. Hybrid models that integrate biomarker signals with symptom-based phenotyping may enable simultaneous classification and mechanistic stratification.^24^^,^^30^, ^31^, ^32^, ^33^, ^34^, ^35^

Strengths and limitations: Strengths include a strict FM-only comparator definition and incorporation of medication burden derived from free-text medication reporting. Limitations include cross-sectional design, reliance on routine clinical labels, pattern-based medication parsing (which may miss uncommon medications or spelling variants), and incomplete questionnaire and SF-36 completion in LC/PASC. Because prior SARS-CoV-2 infection was not systematically assessed in the FM group, some misclassification of post-COVID symptom syndromes as FM cannot be excluded, which may have increased observed similarity between groups. Missing questionnaire data in the LC/PASC cohort represent an additional limitation. Because completion varied across instruments, analyses relied on variable-specific available n. If participants with greater fatigue, cognitive dysfunction, or functional limitation were less likely to complete certain measures, LC symptom and function estimates may be biased toward underestimation. Although available comparisons did not suggest large systematic differences, the modest LC sample size limits definitive conclusions, and future prospective cohorts should incorporate standardized, complete instrument capture. Future work should expand LC sampling, incorporate detailed medication dose/duration, and prospectively collect symptom, function, physiologic, and biomarker data longitudinally.

## Summary

LC/PASC and FM demonstrate partial overlap on routine clinical questionnaires, with medication burden ‒ particularly centrally acting agent exposure ‒ an important contributor to phenotype interpretation. Incomplete SF-36 capture in LC/PASC supports descriptive functional comparisons in real-world datasets. These findings strengthen the rationale for objective biomarkers to improve diagnostic and mechanistic precision.

## Ethics approval

The study was approved by the University of Texas at Austin Institutional Review Board (IRB #2020030008) and conducted in accordance with the Declaration of Helsinki. All participants provided informed consent.

## Funding

This research was funded by the 10.13039/100000002National Institutes of Health, grant number NIH R61NS117211 (KVH) and GR122808 (LRS).

## Data availability statement

The datasets generated and/or analyzed during the current study are available from the corresponding author upon reasonable request.

## Declaration of competing interest

The authors declare no conflicts of interest.

## Appendix Supplementary materials

Image, application 1

## Footnotes

Supplementary material associated with this article can be found, in the online version, at doi:10.1016/j.clinsp.2026.101046.
