# Supplementary material for: Symptom, functional, and medication overlap between long COVID and fibromyalgia
Source: Clinics (Sao Paulo). 2026 Jul 3;81:101046. doi: 10.1016/j.clinsp.2026.101046 (PMC13355519; doi:10.1016/j.clinsp.2026.101046)
Supplement: Supplementary file 1 [file mmc1.docx]

**CLINICS-D-26-00163_ Supplementary Material**

**Supplementary Table S1** Clinical characteristics, symptom burden, and medication burden (LC/PASC vs. FM-only; FM first visits only).

| **Measure** | **LC/PASC** | **FM-only (first visits)** | **SMD (LC-FM)** |
| --- | --- | --- | --- |
| Age, years | 48.4 ± 14.1  (n = 52) | 42.1 ± 13.9  (n = 590) | 0.45 |
| Female, n (%) | 40/54 (74.1%) | 548/591 (92.7%) | -0.50 |
| BMI, kg/m² | 29.7 ± 8.9  (n = 42) | 31.1 ± 8.9  (n = 566) | -0.15 |
| FIQR (or SIQR-equivalent) | 33.0 ± 20.6  (n = 42) | 52.9 ± 19.2  (n = 492) | -1.00 |
| Beck Depression Inventory (BDI) | 14.2 ± 5.9  (n = 43) | 20.3 ± 10.5  (n = 481) | -0.71 |
| Central Sensitization Inventory (CSI) | 55.1 ± 16.9  (n = 43) | 62.2 ± 14.3  (n = 477) | -0.45 |
| McGill Pain Questionnaire (MPQ) | 83.4 ± 51.8  (n = 43) | 92.8 ± 46.7  (n = 475) | -0.19 |
| VAS pain (0–10) | 3.4 ± 1.9  (n = 43) | 5.7 ± 2.1  (n = 576) | -1.17 |
| Total medications identified (free-text) | 1.4 ± 1.5  (n = 54) | 2.2 ± 1.4  (n = 591) | -0.53 |
| Centrally acting medication classes (count) | 1.2 ± 1.4  (n = 54) | 1.9 ± 1.3  (n = 586) | -0.54 |
| Any centrally acting agent, n (%) | 38/54 (70.4%) | 511/586 (87.2%) | -0.41 |
| ≥2 centrally acting agents, n (%) | 21/54 (38.9%) | 349/586 (59.6%) | -0.41 |

**Supplementary Table S2** Comparison of LC/PASC participants with complete versus incomplete SF-36 data.

| **Measure** | **Complete SF-36**  **(n = 43)** | **Incomplete SF-36**  **(n = 11)** |
| --- | --- | --- |
| Age, years | 51.9 ± 12.0  (n = 42) | 42.9 ± 14.2  (n = 10) |
| Female, n (%) | 27/42 (64.3%) | 7/11 (63.6%) |
| BMI, kg/m² | 30.6 ± 9.5  (n = 31) | 25.7 ± 4.7  (n = 11) |
| FIQR/SIQR-equivalent | 48.6 ± 18.1  (n = 36) | 32.4 ± 27.0  (n = 6) |
| Beck Depression Inventory (BDI) | 19.0 ± 10.2  (n = 43) | Not available |
| Central Sensitization Inventory (CSI) | 54.8 ± 16.4  (n = 43) | Not available |
| McGill Pain Questionnaire (MPQ) | 65.3 ± 37.7  (n = 43) | Not available |
